# Impact of a multimodal health education combined with teach-back method on self-management in hemodialysis patients: A randomized controlled trial

**DOI:** 10.1097/MD.0000000000039971

**Published:** 2024-12-27

**Authors:** Yan Liu, Xi Luo, Xue Ru, Caijin Wen, Ning Ding, Jing Zhang

**Affiliations:** a School of Nursing, North Sichuan Medical College, Nanchong City, Sichuan Province, China; b Nursing Department of the Affiliated Hospital of Panzhihua University, Sichuan Province, China; c Department of Nephrology, Affiliated Hospital of Panzhihua University, Sichuan Province, China.

**Keywords:** health literacy, hemodialysis, multimodal health education, nursing care, self-management, teach-back method

## Abstract

**Background:**

To explore the impact of multimodal health education combined with the teach-back method in the self-management of hemodialysis patients.

**Methods:**

Using the convenience sampling method and random number table method, 112 patients who received treatment in the hemodialysis center of a tertiary hospital in Sichuan Province from January 2023 to October 2023 were selected and divided into a control group (n = 56) and an experimental group (n = 56). The control group was given routine care and traditional health education for maintenance hemodialysis patients, of which 31 were male and 25 were female; the average age was (56.04 ± 11.26) years old. The experimental group was given multimodal health education combined with the teach-back method based on the control group, of which 37 cases were male and 19 cases were female; the average age was (53.71 ± 12.72) years old. The changes in self-management score, quality of survival score, and health literacy score were compared between the 2 groups.

**Results:**

Before the intervention, the differences in self-management scores, survival quality scores, and health literacy scores between the 2 groups were not statistically significant (*P* > .05). After the intervention, the total self-management score (96.91 ± 3.02) and the total survival quality score (96.59 ± 4.27) of the experimental group were higher than those of the control group, and the difference was statistically significant (*P* < .05); the total health literacy score of the experimental group (80.30 ± 6.11) was higher than those of the control group, and the difference was statistically significant (*P* < .05); in which, the willingness of financial support of the experimental group score (3.27 ± 4.13) improved, but the difference was not statistically significant (*P* > .05).

**Conclusion:**

Multimodal health education combined with the teach-back method can effectively enhance the self-management level and health knowledge of hemodialysis patients, improve the quality of patients’ survival and disease prognosis, and improve the level of patients’ disease health literacy.

## 1. Introduction

In recent years, chronic kidney disease has become a public health problem of global concern.^[1]^ Maintenance hemodialysis (MHD) is the main alternative therapy for the treatment of end-stage renal disease and has been widely used in clinical practice.^[2]^ According to 2017 U.S. renal data, more than 80% of kidney failure patients worldwide rely on hemodialysis for life support, with China ranking first in the world.^[3]^ According to the latest data from the Chinese Renal Date System, by the end of 2023, the number of dialysis patients in China has exceeded 1 million cases, of which the number of patients relying on hemodialysis to sustain their lives has reached 916,000. It is estimated that the number of dialysis patients in China will continue to grow,^[1]^ and this group has now gradually become a hotspot of concern in Chinese society.

According to self-management theory, self-management behaviors can be explained as a series of protective behaviors adopted by patients in their daily lives to promote health and control disease progression.^[4]^ Some studies^[5]^ have shown that better self-management skills can significantly improve patients’ ability to cope with their illnesses, promote changes in their health behaviors, reduce the burden of disease, and improve the quality of survival. However, most patients lack sufficient knowledge of dialysis-related knowledge, making it difficult to maintain good dialysis behavior during long-term hemodialysis, increasing the disease burden and seriously affecting patients’ quality of life. Therefore, it is crucial to improve the self-management ability of hemodialysis patients.

Health education for hemodialysis patients is still a traditional education model, mainly based on one-way instillation by healthcare personnel or distribution of paper books, etc. The education is in a single form, subject to the limitations of time and space, lack of communication and interaction with the patients, and poor relevance and practicability^[6]^; the model focuses on the general dissemination of information and is unable to assess the degree of understanding of the patient’s knowledge,^[7]^ and fails to meet the diverse information needs of hemodialysis patients.^[6,8]^ The development of modern information technology breaks through the limitations of time and space, and the diversification of information dissemination channels breaks the single mode of traditional health education. The application of multimedia, modern networks, and other technologies, the use of multimode health education methods, teaching content using short videos, pictures, etc., to facilitate dialysis patients and their families to choose the fragmented time for learning, to improve the knowledge rate of the disease of dialysis patients to provide a feasible solution.^[8]^ Several studies have shown^[9–11]^ that multimodal health education helps improve chronic disease patients’ psychological state and self-management ability. Yao et al^[12]^ results show that multimodal health education helps to improve the self-efficacy, treatment adherence, and psychological tolerance of hemodialysis patients. However, some patients forget 40% to 80% of the information immediately after receiving health education, and another part of the information that patients have may be 50% wrong.^[9]^

The teach-back method means that after health education is given to the patient, the patient is asked to express the reception of the health information in his/her own words. In case the information is unclear, the researcher has to repeat the explanation until the patient correctly understands the information he/she has learned.^[13]^ Some studies^[14,15]^ indicated that the teach-back method is a novel and effective education strategy that helps patients retain their knowledge better. The teach-back method has strong universality and has been widely used in the fields of healthcare professions and chronic disease health education.^[16]^ Currently, the application in China is in its infancy, and no study combines multimodal health education and teach-back method, so this study applies multimodal health education and teach-back methods to hemodialysis groups, and explores the effects of the combined intervention of the 2 methods on the hemodialysis patients’ self-management ability, the quality of survival level, and the level of health literacy.

## 2. Methods

### 2.1. Participants

This study was a prospective, 2-arm, unblinded randomized controlled trial. Between January 2023 and October 2023, 112 hemodialysis patients who met the inclusion and exclusion criteria were selected from the hemodialysis center of a tertiary hospital in Sichuan Province, China. Participants were considered if they met the following criteria: age ≥18 years, clear consciousness and normal language expression ability; meeting the diagnostic criteria of chronic kidney disease and MHD-related therapeutic indications^[1]^; stable condition, and the duration of dialysis for MHD was >3 months, regular dialysis 2 to 3 times per week, 4 to 5 h/session; and patients’ informed consent to this study. Participants were excluded if they transferred out, had a kidney transplant, were on peritoneal dialysis, died, and were lost to follow-up.

The sample size of this study was estimated by PASS 15.0 software. According to the results of similar studies, the mean score of the self-management questionnaire increased by 12.30 after the intervention, with an overall standard deviation of about 4.15 (*α* = 0.05, *β* = 0.09). It was calculated that 92 patients were included in this study. Taking into account the 20% attrition rate, the sample size was finally expanded to 120 patients, but 8 participants were excluded due to language barriers, illnesses, etc so the final sample size of this study was 112 participants (participation rate = 93.3%).

### 2.2. Interventions

In this study, 112 patients were randomly assigned to either the intervention group or the control group based on a 1:1 ratio, with 56 patients in each group. The random assignment sequence was generated by research assistants not involved in this study using a web-based random sequence generator (https://www.random.org/) and permutation randomized block group assignment. The control group received traditional health education, and the experimental group implemented multimodal health education combined with the teach-back method based on the control group, and the intervention time was 3 months, as follows:

#### 2.2.1. Control group

The control group adopts traditional health education such as oral explanation, written teaching, and centralized education, as follows:

Oral explanation: the research assistant who did not participate in the study informed the patients and their families about the knowledge of hemodialysis-related diseases and the basics of hemodialysis nursing and taught them how to maintain the dialysis line. Written preaching: paper materials were distributed and patients were encouraged to ask questions actively after reading and answering them. Centralized education: three hemodialysis knowledge lectures were given, with a duration of 1.0 to 1.5 hours. The content of the education was the same topic as the multimodal health education of the experimental group: health guidance; dietary guidance; interpretation of laboratory test slips; self-testing of vascular access; exercise guidance; medication guidance; and prevention and treatment of complications.

#### 2.2.2. Experimental group

(1) Establishment of the intervention team: 10 members in total, 1 nurse manager, 5 national blood purification specialist nurses, 1 doctor, 1 medical information technology personnel, and 2 postgraduate students. The members of the team were uniformly trained in the theory related to the teach-back method and the specific implementation skills, and they could only participate in this study after passing the examination.
(2) Multimodal health education: 2 specialized nurses and 1 nephrologist served as the main implementers of health education. Multimode health education was implemented in the following ways:
  ① Knowledge explanation: the researcher used oral explanation combined with written education to provide one-on-one knowledge explanation to patients, the lecture time is 30 to 40 minutes, and the content of the lecture is the same as that of the control group.
  ② Network online learning: through the online platform to push hemodialysis disease-related articles, and text, pictures, and animation to assist in the form of learning; daily timed push short video, through the online platform to send 1 to 2 short videos on hemodialysis topics, the duration of 10 to 15 minutes. And set up a network sign-in applet to supervise patients’ learning (Table 1).
  ③ Individual guidance: regularly answering questions by the researcher to hemodialysis patients face-to-face or through the online platform for 20 to 30 minutes every weekend.
  ④ Follow-up visit: follow-up visit by phone, 1 time/mo, to strengthen health education and guidance.
(3) Teach-back method of health education:

The teach-back method was used throughout the entire process of health education for patients in the experimental group in 4 steps, that is, explanation, repetition, assessment and correction, and reassessment.

① Health education: the researcher explained or demonstrated the knowledge related to hemodialysis to the patients according to the health education program that had been developed, and instructed the patients to learn online through the Internet.
② Repetition of knowledge: at the end of the lecture, the patients retell the health education content they have mastered in their language. The researcher should pay attention to creating a relaxed and pleasant learning atmosphere in this process to prevent patients from psychological discomfort.
③ Assessment and correction: after the patient retells the information or at the end of the questioning, the researcher assesses the patient according to the patient’s retelling of the information to understand the patient’s understanding and mastery of health knowledge. For the content that the patients did not understand or did not understand properly, the health knowledge was explained to the patients again, and if the patients still could not understand correctly, the health education method was changed to ensure that the patients could understand correctly.
④ Ask questions again and evaluate: at the end of the study, ask patients open-ended questions, such as “According to your actual situation, how will you carry out the daily care of the hemodialysis line?” If the patient can answer correctly and comprehensively, it means that the patient has mastered the knowledge and the health education is over; if not, repeat steps ① to ④ until the patient has fully mastered it.

**Table 1:** Content pushed by health education mode.

Push time	Theme	Video playback content
Weeks 1–3	Health guide	① The importance of healthy behaviors for patients with MHD. ② The introduction of kidney function and the factors affecting its injury. ③ Dialysis-related knowledge popularization. ④ The importance of mental adjustment. ⑤ Patient health education and self-management guidance.
Weeks 4–6	Dietary guidance	① Principles of nutritional dietary intake, and nutritional treatment. ② Reasonable control of water and ion intake. ③ Common food ingredient inquiry form. ④ The danger of malnutrition. ⑤ Taboo and cautious food.
Weeks 7–9	Laboratory interpretation	① Hemodialysis commonly used laboratory indicators and inspection frequency. ② Data analysis of anemia in hemodialysis. ③ Quarterly analysis of laboratory indicators. ④ Monitoring of various indexes of sodium, calcium, and phosphorus.
Weeks 10–13	Self-monitoring of vascular access	① Precautions after the operation of new arteriovenous fistula. ② How to maintain arteriovenous fistula unimpeded. ③ Self-monitoring of internal arteriovenous fistula. ④ Guidance before and after dialysis.
Weeks 14–16	Sports instruction	① Improve patients’ awareness of exercise self-management and health-related lifestyle. ② The choice of exercise mode, duration, and frequency. ③ Self-monitoring of movement center rate.
Weeks 17–20	Medication guidance	① The selection and precautions of commonly used drugs. ② Drug use guidelines. ③ Monitoring and treatment of adverse drug reactions.
Weeks 21–24	Complications and management	① Common dialysis-related complications and prevention. ② Abnormal situation and prevention of complications of arteriovenous fistula. ③ Risk and treatment of abnormal indexes of sodium, calcium, and phosphorus.
Every Sunday	Doctor-patient interaction	Researchers and patients interact through the network platform to answer questions from patients.

### 2.3. Research tools

#### 2.3.1. General characteristics of participants

The study used self-designed demographic and clinical information forms, the Hemodialysis Self-Management Behaviour Scale, the World Health Organization Quality of Survival Measurement Scale Short Form, and the Chronic Disease Health Literacy Scale for data collection. Demographic information (including patient age, gender, education, employment status, income, marital status, and health insurance status) was collected at baseline. And clinical information (dialysis duration, dialysis frequency, and complications).

#### 2.3.2. Hemodialysis Self-management Behavior Scale

The self-management behavior questionnaire for hemodialysis patients developed by Chinese scholars Wang et al^[17]^ was used, which consisted of 25 entries in 5 dimensions, that is, eating and drinking volume management behavior, exercise hobby behavior, fluid, and ion restriction behavior, general status management, and psychosocial behavior, and was scored on a Likert 4-point scale with scores ranging from 25 to 100, with the higher scores indicating the higher level of self-management behavior of the patients. The Cronbach alpha coefficient for internal consistency of the Chinese version of the questionnaire was 0.812, and the Cronbach alpha coefficients for the dimensions were distributed from 0.716 to 0.826. In this study, the Cronbach alpha coefficient was 0.867.

#### 2.3.3. Chinese version of the World Health Organization Quality of Life Measurement Scale brief table

The Chinese version of the World Health Organization Quality of Life Measurement Scale Brief Form consists of 26 entries, of which 2 measures of overall quality of life are independently analyzed, and the other 24 entries can be divided into 4 dimensions: physiological domains, psychological domains, social relationship domains, and environmental domains; a Likert 5-point scale was used, and the higher the reverse scoring scores of some of the entries, the better the dimensional the better the functioning of the dimension.^[18]^ The overall Cronbach alpha coefficient of the Chinese version of the scale was 0.94, and the Cronbach alpha coefficients of the dimensions were all >0.75 except for the domain of social relations, which were 0.76, 0.88, 0.67, and 0.89, respectively, which showed good internal consistency of the scale. In this study, the Cronbach alpha coefficient was 0.833 for the summary scale and 0.762 to 0.812 for each dimension.

#### 2.3.4. Chinese version of Chronic Disease Health Literacy Scale

The Chronic Disease Health Literacy Scale was adopted from the Chinese version that was Chineseised and revised by Chinese scholars Sun et al^[19]^ based on the HeLMS scale.^[20]^ The scale consists of 24 items in 4 dimensions, that is, information acquisition ability, communication and interaction ability, willingness to improve health, and willingness to provide financial support. All of them were rated on a 5-point Likert scale with a score of 1 to 5. The total score is 24 to 120 points the higher the score the higher the health literacy level of the subjects. The overall Cronbach alpha coefficient for the Chinese version of the scale was 0.901, with good internal consistency. In this study, the Cronbach alpha coefficient was 0.839.

### 2.4. Data collection and outcome

In this study, the level of self-management, the level of quality of survival, and the level of health literacy of patients in both groups were measured by the study leader through a face-to-face questionnaire at (baseline) T0 and (3 months) T1, respectively. Demographic and clinical data of the participants in both groups were retrieved from e-cases.

### 2.5. Data analysis

IBM SPSS version 27.0 was used for data analysis. PASS version 15.0 was used to estimate the sample size. Frequencies and percentages were used to describe categorical variables. Mean ± standard deviation was used to describe continuous variables. Independent *t* tests and chi-square tests were used to compare the homogeneity of demographic and clinical characteristics as well as baseline outcome variables between the 2 groups, with the inclusion of control variables (age, age on dialysis, etc). All statistical tests were performed using 2-sided tests, and a *P* value of <.05 (two-sided) was statistically significant.

### 2.6. Ethics and morality

The study was conducted by the Declaration of Helsinki (revised 2013). The study was approved by the Medical Ethics Committee of the Affiliated Hospital of Panzhihua College (No. KY20230701). An informed consent form was provided to the patients for this study and stated that participation was voluntary and that patients could withdraw from the study at any time without affecting the treatment. Data collection methods and confidentiality requirements were explained to patients before signing the informed consent form. All study data were stored on a password-protected external hard drive and kept by research assistants who were not involved in this study.

## 3. Results

### 3.1. Participants’ general characteristics

**Figure 1.**
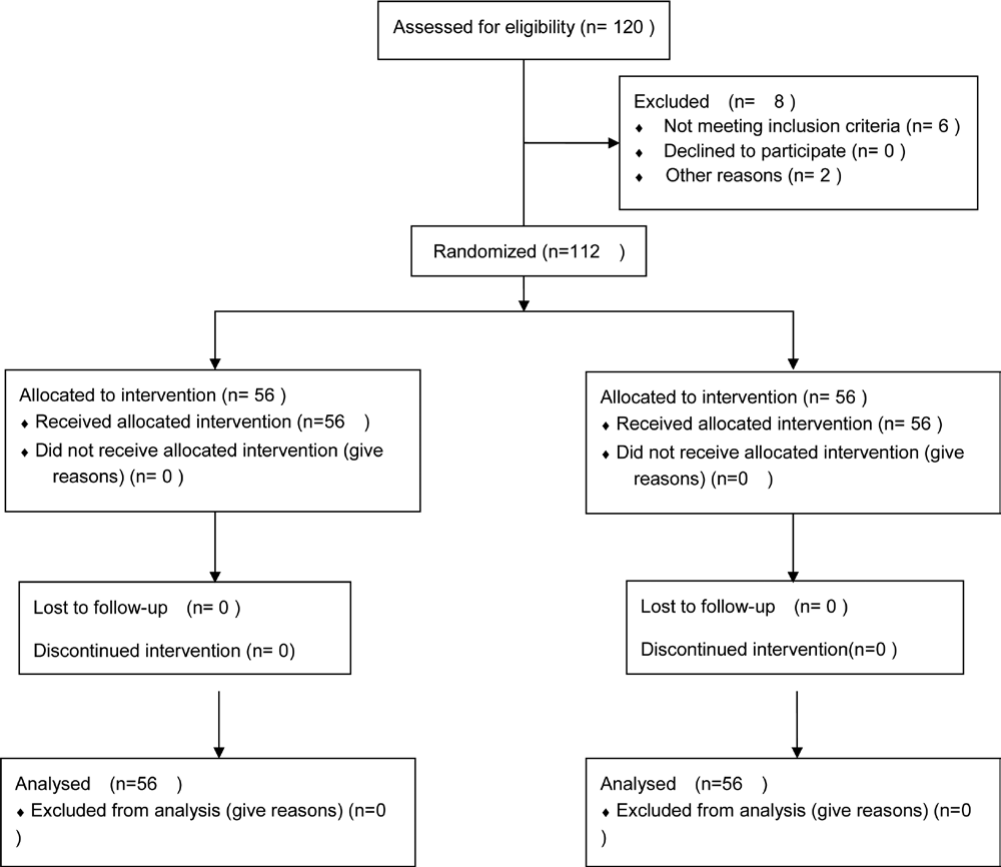
CONSORT flow diagram for participants in the study.

Of the 120 patients recruited between January 2023 and October 2023, 112 completed the study (participation rate = 93.3%), including 56 patients in both the control and experimental groups. Eight patients were excluded from the final data due to language barriers (6) or illness (2) (Fig. 1).

Among them, 68 (60.0%) were males and 44 (39.2%) were females. The average age was 54.88 ± 12.01 years. Thirty-one (27.6%) cases were in primary school and below, 29 (25.8) cases were in junior high school, 30 (26.7%) cases were in high school/secondary school, and 22 cases (19.6%) were in tertiary school and above; 36 patients (32.1%) had a duration of dialysis of <1 year, 50 (44.6%) patients had a duration of dialysis of 1 to 5 years, and 26 (23.2%) patients had a duration of dialysis of >5 years. A comparison of the general information between the control group and the experimental group is shown in Table 2.

**Table 2:** General data ratio of maintenance hemodialysis patients between the 2 groups [example (percentage, %)].

Item	Control group (n = 56)	Experimental group (n = 56)	*X*^2^/*t* value	*P* value
Age	56.04 ± 11.26	53.71 ± 12.72	1.022†	.309
Sex				
Male	31 (55.4)	37 (66.1)	1.348‡	.246
Female	25 (44.6)	19 (33.9)		
Educational background				
Primary and below	17 (30.4)	14 (25.0)	1.868‡	.600
Junior high school	12 (21.4)	17 (30.4)		
High school/technical secondary school	17 (30.4)	13 (23.2)		
College or above	10 (17.9)	12 (21.4)		
Dialytic year				
<1 yr	15 (26.8)	21 (37.5)	2.385‡	.304
1–5 yr	25 (44.6)	25 (44.6)		
>5 yr	16 (28.6)	10 (17.9)		
Matrimony				
Have a spouse	43 (76.8)	41 (73.2)	0.190‡	.663
No dubbing	13 (23.2)	15 (26.8)		
Occupation				
Be on the job	22 (39.3)	25 (44.6)	0.330‡	.566
On the job	34 (60.7)	31 (55.3)		
Number of primary diseases				
0	12 (23.3)	7 (12.5)	3.352‡	.187
1	31 (55.4)	28 (50.0)		
≥2	13 (23.3)	21 (37.5)		
Per capita monthly household income				
<3000	7 (12.5)	2 (3.6)	4.787‡	.091
3000–5000	30 (53.6)	26 (46.4)		
>5000	19 (33.9)	28 (50.0)		

† Paired *t* test.

‡χ ^2^ test.

### 3.2. Comparison of self-management behavior scores of hemodialysis patients in 2 groups

Before the intervention, there was no significant difference between the total score of self-management behavior and the scores of each dimension in the 2 groups of hemodialysis patients (*P* > .05). After 3 months of intervention, the total score of self-management behavior and the scores of each dimension in the experimental group were higher than those in the control group, and the difference was statistically significant (*P* < .05) (Table 3).

**Table 3:** Comparison of self-management behavior scale scores between the 2 groups of maintenance hemodialysis patients before and after intervention (score, $\bar{x}\pm \;s$).

Variables	Baseline				After 3 mo of intervention			
	Control group	Experimental group	*t* value	*P* value	Control group	Experimental group	*t* value	*P* value
Diet management	14.70 ± 1.57	15.07 ± 0.82	−1.580	.118	15.16 ± 1.09	18.50 ± 0.93	−8.998	<.001**
Sports management	16.96 ± 2.14	17.07 ± 1.62	−0.298	.766	17.61 ± 1.26	19.54 ± 0.76	−9.800	<.001**
Liquid and ion intake management	31.20 ± 2.69	32.00 ± 2.69	−1.767	.080	31.95 ± 2.69	35.46 ± 1.16	−8.998	<.001**
General state management	18.45 ± 1.79	18.73 ± 1.71	−0.861	.391	18.71 ± 1.93	23.41 ± 0.82	−16.721	<.001**
Self−management score	81.30 ± 5.10	82.88 ± 3.56	−1.891	.062	83.43 ± 3.45	96.91 ± 3.02	−21.95	<.001**

*P < .05

** *P* < .001.

### 3.3. Comparison of quality of life measurement scores in 2 groups of hemodialysis patients

Before the intervention, there was no statistically significant difference between the total quality of life scores and the scores of each dimension of the hemodialysis patients in the 2 groups (*P* > .05). After 3 months of intervention, the total score of quality of life and the scores of each dimension in the experimental group were higher than those in the control group, and the difference was statistically significant (*P* < .05) (Table 4).

**Table 4:** Comparison of WHOQOL-BREF scale scores between the 2 groups of maintenance hemodialysis patients before and after intervention (score, $\bar{x}\pm \;s$).

Variables	Baseline				After 3 mo of intervention			
	Control group	Experimental group	*t* value	*P* value	Control group	Experimental group	*t* value	*P* value
Physiological field	22.50 ± 1.56	22.86 ± 2.11	−1.016	.312	22.50 ± 3.18	23.98 ± 2.41	−2.774	.007*
Psychological field	18.66 ± 1.55	19.02 ± 1.27	−1.332	.186	19.14 ± 2.27	22.46 ± 1.78	−8.625	<.001**
Social relations field	9.48 ± 1.26	9.89 ± 1.06	−1.865	.065	9.64 ± 1.05	10.57 ± 1.14	−4.476	<.001**
Environmental field	28.91 ± 3.41	28.21 ± 3.29	1.098	.275	29.86 ± 1.79	32.27 ± 1.80	−7.092	<.001**
Subjective perception of quality of life	3.13 ± 0.69	3.20 ± 0.61	−0.578	.564	3.34 ± 0.58	3.66 ± 0.48	−3.198	.002*
Subjective perception of health status	3.05 ± 0.75	3.27 ± 0.65	−1.621	.108	2.84 ± 0.733	3.64 ± 0.48	−6.848	<.001**
WHOQOL-BREF total score	85.73 ± 4.94	86.45 ± 4.72	−0.802	.424	87.32 ± 6.01	96.59 ± 4.27	−9.400	<.001**

* *P* < .05

** *P* < .001.

WHOQOL-BREF = Chinese version of the World Health Organization Quality of Life Measurement Scale brief table.

### 3.4. Comparison of health literacy scores of hemodialysis patients in 2 groups

Before the intervention, there was no statistically significant difference between the health literacy of the 2 groups of hemodialysis patients (*P* > .05). After 3 months of intervention, the health literacy scores of both groups were higher than before intervention, and the total score obtained by the experimental group was higher than that of the control group (*P* < .05), in which the scores of information acquisition ability, communication and interaction ability, and willingness to improve health were higher than that of the control group, with statistically significant differences (*P* < .05), and the willingness of economic support was not statistically significant (*P* > .05) (Table 5).

**Table 5:** Comparison of health literacy Scale scores between the 2 groups of maintenance hemodialysis patients before and after intervention (score, $\bar{x}\pm \;s$).

Variables	Baseline				After 3 mo of intervention			
	Control group	Experimental group	*t* value	*P* value	Control group	Experimental group	*t* value	*P* value
Information acquisition ability	11.84 ± 2.68	10.93 ± 2.57	1.834	.069	12.14 ± 1.62	30.27 ± 2.72	−29.285	<.001**
Communication and interaction ability	17.02 ± 4.67	18.27 ± 1.90	−1.855	.066	18.04 ± 4.31	30.34 ± 2.52	−18.404	<.001**
Intention to improve health	6.96 ± 2.89	7.05 ± 2.29	−0.181	.856	7.70 ± 1.14	16.43 ± 2.32	−25.278	<.001**
Willingness to support the economy	2.64 ± 0.94	2.66 ± 0.94	−0.100	.920	2.59 ± 0.57	3.27 ± 4.13	−1.219	.285
Health literacy score	38.46 ± 6.10	38.91 ± 4.37	−0.445	.657	40.46 ± 4.81	80.30 ± 6.11	−38.32	<.001**

*P < .05

** *P* < .001.

## 4. Discussion

Hemodialysis treatment can effectively eliminate toxins in patients with chronic renal failure, slow down the disease process, and prolong the survival time of patients, but due to the long-term treatment of the patient’s daily life has a certain impact on the patient’s daily life, reducing the treatment compliance, aggravating the progress of the disease, which will make the patient pessimism, depression, and other negative emotions, affecting the patient’s mental health and reducing the quality of life. Therefore, giving effective interventions plays an important role in the self-management ability of hemodialysis patients.^[21]^ A study^[4]^ showed that self-management is a predictor of disease prognosis. Therefore, it is extremely important to intervene with hemodialysis patients to improve their self-management skills.^[22]^ In this study, a multimodal health education combined with a teach-back method was used to intervene with patients and achieved significant results.

### 4.1. The effect of multimodal health education combined with teach-back method on the self-management ability of hemodialysis patients

The scores and total scores of the 4 dimensions of dietary behavior management, exercise behavior management, fluid and ion intake management, and general status and psychosocial management in the experimental group were significantly higher than those of the control group, and the difference was statistically significant (*P* < .05). Wan et al^[5]^ and Kim et al^[23]^ showed that the Internet-based health education model helps to improve patients’ self-management; the results of Oh et al^[24]^ showed that the teach-back method can effectively improve the self-care ability of heart failure patients. This is consistent with the results of the present study. In the current study, patients who received multimodal health education combined with teach-back method showed significant improvement in self-management (*P* < .05), and the reasons for this were analyzed as follows: traditional health education is mostly based on simple theoretical instructions, lacks intuition and vividness, and the degree of knowledge comprehension is easily affected by the patient’s age, literacy level, and health literacy,^[25]^ which leads to the fact that health knowledge is easy to be forgotten, and thereby health behavior in family care failed to put into action or difficult to adhere to or, so the educational effect of the traditional health education model is poor. In the implementation of the program, the traditional health education content is combined with multimode health education, the content is intuitive and vivid, doctor-patient interaction is achieved through the use of modern network information platforms, and the team members carry out personalized nursing interventions for different patients’ conditions, to increase the information support for MHD patients, which is conducive to stimulating the subjective initiative of the patients, and to improve the self-management ability of MHD patients. Several studies have shown^[26,27]^ that the teach-back method can deepen patients’ understanding of the disease. A meta-analysis^[28]^ showed that the teach-back method was significantly and positively correlated with self-efficacy. In this study, the teach-back method was used throughout the entire health education process of hemodialysis patients, in which the patients’ mastery of the health education content was assessed through the combination of health knowledge lectures and open-ended questions, and the ambiguous or inaccurate knowledge of the patients was repeated, and then the mastery of the patients was assessed again at the end. This repetition process can effectively deepen the patients’ understanding of health knowledge, enhance the patients’ self-esteem, self-confidence, and subjective initiative, promote the change in patients’ health behavior, effectively improve the therapeutic effect of hemodialysis, and enhance the patients’ self-management ability.

### 4.2. The effect of multimodal health education combined with teach-back method on the quality of survival of hemodialysis patients

The ultimate goal of implementing health interventions for MHD patients is to improve their self-management ability by meeting their health needs, thereby improving the quality of survival and realizing positive recovery.^[29]^ Compared with the control group after the intervention, the scores and total scores of the experimental group in the 6 dimensions of physiological domain, psychological domain, social relationship domain, environmental domain, subjective feelings about the quality of their existence, and subjective feelings about their health status were significantly higher than those of the control group, and the difference was statistically significant (*P* < .05). The results of several studies have shown^[26,29–31]^ that the application of the teach-back method can effectively improve patients’ ability to care for themselves and their quality of life; in addition, the study of Ghanbari et al^[32]^ showed that health education based on the teach-back method significantly improved treatment adherence among dialysis patients with end-stage renal disease. Scholars such as Ghorbani et al^[7]^ stated that the intervention of using the teach-back method based on the health education approach The longer the duration was more beneficial to the patients. These findings are consistent with the results of the present study. The reasons are as follows: teach-back method is different from the unidirectional output of traditional health education, and pays more attention to the 2-way transmission of information. Through the explanation of health education content and the interaction of health care personnel, as well as the assessment and feedback on the patient’s level of comprehension, it is more capable of carrying out targeted and individualized education for the patients, to deepen the patient’s knowledge of the disease and understanding of hemodialysis, and to reduce the risk of the patients’ forgetfulness of the information and their negative emotions such as the fear of the progression of the disease. The risk of forgetting information and the fear of disease progression are reduced. The teach-back method is more applicable and can be used for patients with low literacy or limited literacy, which can deepen MHD patients’ understanding of health information, have a positive effect on disease cognition, disease behavior, and disease beliefs, and help to enhance patients’ self-confidence in their disease, promote social participation, and improve the quality of survival and disease prognosis. Due to the long duration and cyclical nature of hemodialysis treatment, patients can repeat their learning during hemodialysis treatment, thus increasing medical support for patients, helping to improve their treatment adherence and their quality of life, and benefiting them.

### 4.3. The effect of multimodal health education combined with teach-back method on health literacy of hemodialysis patients

After 3 months of intervention, the scores and total scores of the experimental group were significantly higher than those of the control group in the 3 dimensions of information acquisition ability, communication and interaction ability, and willingness to improve health, and the difference was statistically significant (*P* < .05). Sophie et al^[32]^ showed that the teach-back method was effective in improving the patient’s knowledge of the disease. Ren’s^[33]^ study pointed out that health knowledge is an important disease decision-making factor. The study by Qiong^[34]^ showed that the health education model based on the Internet platform could effectively improve the health knowledge of urban elderly, similar to the results of this study. Hemodialysis treatment is a continuous process, and patients tend to take medication according to their current situation during the treatment period,^[35]^ but due to the lack of disease knowledge of the majority of patients and their families, they are unable to take medication reasonably according to their situation, which aggravates the disease burden of the patients and affects the prognosis of the disease. One study^[36]^ showed that health literacy is an independent determinant of disease outcome, the level of health literacy of patients is closely related to disease prognosis, and the higher the level of health literacy, the better the prognosis of the disease. In addition, a study^[37]^ showed that an intervention using a combination of multimedia and teach-back methods helped to improve the health literacy of diabetic patients. It is consistent with the results of this study. The reasons were analyzed as follows: previous studies only used a single health education model, that is, teach-back method or multimodal health education model, to deliver a large amount of knowledge,^[7]^ which was unable to assess the disease knowledge rate of the patients and could not ensure the effectiveness of health education. Therefore, this study combines multimodal health education with the teach-back method, breaks the traditional single-mode health model, breaks through the 1-lesson, 1-lecture education method, and utilizes the modern information network platform to deliver health education tutorials to patients in a variety of ways, such as short videos, pictures, and on-site explanations. In this study, the multimode health education joint teaching back method reasonably exerts the advantages of both, providing a convenient and fast platform for patients using the network in the research process, making it easier for patients to learn, combining the role of the network to make health education more interesting, and easier for patients to understand and master. During the study, the continuous use of “explanation-assessment-clarification-reassessment” and multimodal health education enhances the memory of health knowledge of hemodialysis patients, reduces the risk of forgetting the information, improves the patients’ knowledge of the disease, and effectively improves the patients’ self-management ability and health literacy. Since medication regimen is an important factor in the treatment of hemodialysis patients, the support of information resources in patients’ health education can help to improve patients’ information health literacy and improve their treatment adherence.

In addition, this study is also consistent with the findings of Chinese scholar Zhang et al.^[38]^ It is known from the previous survey study that the score of economic support willingness of hemodialysis patients was improved but not significant and not statistically significant (*P* > .05). It is because the willingness of economic support is related to the patient’s economic situation, family income, and other objective factors, and after 3 months of health education intervention, the economic aspects did not improve much, so the difference is not statistically significant (*P *> .05).

## 5. Limitations

There were several limitations to this study. First, this study was not blinded to participants and interveners because of the nature of the educational intervention; second, this study was not set up with an application to record patients’ online learning or browsing of short videos; in addition, this study relied on patients’ self-assessment reports and did not use an objective measurement tool to monitor the patient’s physical functioning, and thus there is some risk of bias; and lastly, this study’s participant group was selected only from hemodialysis patients at a tertiary care hospital in Sichuan Province, China. Finally, this study only selected hemodialysis patients in a tertiary hospital in Sichuan Province, China, which is a small and regionally homogeneous sample size, limiting the generalizability of the results. Therefore, we suggest that the sample size could be enlarged or a multicenter study could be conducted; an interactive application could be set up to record studies or send reminders during the study; and telemedicine could be applied to the home care of hemodialysis patients by combining the functions of a web-based platform.

## 6. Conclusions

In this study, a multimodal health education based on a web-based platform for information dissemination combined with the teach-back method was effective in improving the self-management ability of hemodialysis patients, prompting changes in health behaviors, improving the quality of life of patients, and improving their health literacy level. This study suggests that the combination of a multimedia social networking platform and the teach-back method can provide a better and more engaging learning experience for patients and help healthcare professionals receive more and faster feedback from patients, which is worth promoting in clinical settings.

## Acknowledgments

The authors thank the doctors and nurses who supported and assisted our study at the Hemodialysis Center of Panzhihua College Hospital. The authors also thank all hemodialysis patients for their cooperation.

## Author contributions

**Conceptualization:** Yan Liu, Jing Zhang.

**Formal analysis:** Yan Liu, Xi Luo.

**Writing—original draft:** Yan Liu.

**Supervision:** Xi Luo, Caijin Wen, Jing Zhang.

**Writing—review & editing:** Xi Luo, Caijin Wen, Ning Ding, Jing Zhang.

**Visualization:** Xue Ru, Ning Ding.

**Software:** Caijin Wen.

**Funding acquisition:** Jing Zhang.
